# Quantification of massively parallel sequencing libraries – a comparative study of eight methods

**DOI:** 10.1038/s41598-018-19574-w

**Published:** 2018-01-18

**Authors:** Christian Hussing, Marie-Louise Kampmann, Helle Smidt Mogensen, Claus Børsting, Niels Morling

**Affiliations:** Section of Forensic Genetics, Department of Forensic Medicine, Faculty of Health and Medical Sciences, University of Copenhagen, DK-2100 Copenhagen, Denmark

## Abstract

Quantification of massively parallel sequencing libraries is important for acquisition of monoclonal beads or clusters prior to clonal amplification and to avoid large variations in library coverage when multiple samples are included in one sequencing analysis. No gold standard for quantification of libraries exists. We assessed eight methods of quantification of libraries by quantifying 54 amplicon, six capture, and six shotgun fragment libraries. Chemically synthesized double-stranded DNA was also quantified. Light spectrophotometry, i.e. NanoDrop, was found to give the highest concentration estimates followed by Qubit and electrophoresis-based instruments (Bioanalyzer, TapeStation, GX Touch, and Fragment Analyzer), while SYBR Green and TaqMan based qPCR assays gave the lowest estimates. qPCR gave more accurate predictions of sequencing coverage than Qubit and TapeStation did. Costs, time-consumption, workflow simplicity, and ability to quantify multiple samples are discussed. Technical specifications, advantages, and disadvantages of the various methods are pointed out.

## Introduction

Massively parallel sequencing (MPS) is widely used in biological research^1–5^. Optimization of the MPS workflow is important for both economic and timewise reasons^6,7^. Maximizing the number of samples that can be sequenced in one experiment is one obvious way of reducing the costs. It requires accurate quantification of each sequencing library and pooling of the libraries in equal molar ratios. If the quantifications underestimate the number of molecules in the libraries, the sequencing experiments may fail completely because the number of molecules per bead or flowcell exceeds the capacity of the sequencing platform. If the library concentrations are overestimated, the sequencing capacity will not be used in an optimal way. If the quantification of some libraries is underestimated and others are overestimated, the sequencing depth (coverage) of the samples will vary. Some samples may have high coverage with many redundant sequencing reads and loss of sequencing capacity as a result. Some samples may have low coverage and sequencing of these samples may have to be repeated because the results of the sequencing analysis are unreliable.

Each library consists of the target sequences flanked by adapter sequences needed for the downstream reactions (clonal amplification and sequencing) and subsequent data analysis (key sequence for quality control and barcodes for identification of the sample)^8–10^. The target sequences are generated by fragmentation, capture protocols, or PCR amplification^11–14^, and the adapters are ligated to the fragments. Ideally, the quantification should differentiate between molecules with target sequences and other molecules, e.g. adapter and primer dimers.

Quantification methods vary in quantitative range, sensitivity, costs, workflow simplicity, etc. Spectrophotometry can be applied for library quantification, either by measuring light penetrance as in e.g. the NanoDrop instrument (Thermo Fisher Scientific, Waltham, MA, USA), or by measuring the fluorescence as in e.g. the Qubit instrument (Thermo Fisher Scientific). Electrophoresis displays the size distribution of the DNA fragments in the library, which makes it possible to distinguish between molecules with target sequences and molecules only comprising adapter or primer dimers^15–17^. Finally, various quantitative PCR (qPCR) assays have been developed for MPS library quantification^15,17,18^. qPCR is highly sensitive and capable of quantifying specific molecules, e.g. molecules with the required adapters in the 5′ and 3′ends of the fragments.

In this study, eight different methods for MPS library quantification were used to quantify PCR amplicon, capture, and shotgun fragment libraries along with chemically synthesized double-stranded DNA (dsDNA) oligos. The concentrations, fragment lengths, adapter dimer abundance, and PCR inhibitor content were investigated.

## Materials and Methods

### Samples, DNA extraction, and library construction

Blood samples from 41 individuals, buccal swabs on Flinders Technology Associates cards (FTA cards) from 15 individuals, and six crime case samples from forensic case work were used (Table 1). DNA from blood samples and DNA from buccal swabs on FTA cards was extracted using the EZ1 DNA Investigator Kit (Qiagen, Hilden, Germany) and the EZ1 advanced XL instrument (Qiagen) as previously described^12^. DNA was extracted from the crime case samples using Chelex 100 (Bio-Rad, Hercules, CA, USA) (DNA from three samples: a toothbrush, nails from a body recovered from the sea, and DNA swabbed from a stick used as toothbrush), EZ1 DNA Investigator (DNA from formalin fixated paraffin embedded tissue), QIAquick (Qiagen) in combination with Amicon Ultra (Merck, Darmstadt, Germany) (DNA from a bone), or phenol/chloroform (DNA from a jaw bone)^19^. The DNA extracted from crime case samples was archived with low TE buffer (10 mM Tris-HCl, 1 mM EDTA), and stored at −20 °C for 2–15 years before this study. The work was approved by the Danish ethical committee (H-4-2011-081). Samples were taken from the biobank of the Department of Forensic Medicine, University of Copenhagen (RIBVF; approved by the Danish Data Protection Agency, j.no. 2002-54-1080). The Danish ethical committee waived the requirement for informed consent (H-4-2011-081). The DNA extracts from the crime case samples were investigated in agreement with Danish criminal law.

**Table 1:** Samples and libraries used in this study.

Library	Samples	Number of samples
HID-Ion AmpliSeq Identity Panel	Buccal swabs on FTA cards	12
HID-Ion AmpliSeq Identity Panel	Forensic crime case samples	6
Ion Xpress Plus Fragment Kit	Buccal swabs on FTA cards	6
NimbleGen SeqCap Target Enrichment	Blood	6
HID-Ion AmpliSeq Ancestry Panel	Blood	35

DNA extracted from 12 buccal swabs and six crime case samples was amplified using the Precision ID Identity Panel (previously called HID-Ion AmpliSeq Identity Panel) (Thermo Fisher Scientific). DNA libraries were constructed using the Ion AmpliSeq Library Kit 2.0 (Thermo Fisher Scientific) according to the manufacturer’s recommendations. The libraries were grouped as either “well amplified”, “adapter dimer rich”, or “PCR inhibited” based on analysis with the 2100 Bioanalyzer using the High Sensitivity DNA Assay (Agilent Technologies, Santa Clara, CA, USA) (Supplementary Fig. 1). Furthermore, three of the six samples categorized as “PCR inhibited” libraries were shown to be PCR inhibited during quantification with the Applied Biosystems 7500 Real-Time PCR System (ABI7500) instrument (Thermo Fisher Scientific) and the Quantifiler Human DNA Quantification Kit (Thermo Fisher Scientific) following the protocols of the manufacturers.

DNA extracted from six buccal swabs was sonicated using the Covaris S220 instrument (Thermo Fisher Scientific) with peak power 175 W, duty factor 10%, 200 cycles per burst, and 120 sec treatment time. DNA libraries were constructed using the Ion Xpress Plus Fragment Kit (Thermo Fisher Scientific) following the manufacturer’s protocol.

DNA extracted from blood samples from six individuals was processed into NimbleGen (Roche, Basel, Switzerland) capture libraries as previously described^11^.

DNA extracted from blood samples from 35 individuals was amplified using the Precision ID Ancestry panel (previously called HID-Ion AmpliSeq Ancestry Panel) (Thermo Fisher Scientific). DNA libraries were constructed using the Ion AmpliSeq Library Kit 2.0 (Thermo Fisher Scientific) following the manufacturer’s protocol with one exception: the number of PCR cycles was increased from 21 to 25 cycles in order to obtain sufficient amounts of PCR products.

### Generation of synthetic dsDNA oligos

Two synthetic dsDNA oligos were designed (DNA Technology, Risskov, Denmark). The Ion Torrent oligo was identical to the “A” and “P1” adapter sequences (Supplementary Table 1). The Illumina oligo was identical to the “i7” and “i5” adapter sequences. Oligo concentrations were measured by the supplier using the SpectraMax Plus 384 Absorbance Plate Reader instrument (VWR International, Radnor, PA, USA).

### Assessment of library quantification methods by quantifying libraries and dsDNA oligos

Precision ID libraries, Ion Xpress fragment libraries, NimbleGen libraries, and dsDNA oligos were quantified using NanoDrop 1000 (Thermo Fisher Scientific), the High Sensitivity Assay with the Qubit 2.0 (Thermo Fisher Scientific), the High Sensitivity Assay with the 2100 Bioanalyzer (Agilent Technologies), the High Sensitivity D1000 ScreenTape Assay with the 2200 TapeStation (Agilent Technologies), the DNA High Sensitivity Assay with the GX Touch (PerkinElmer), and the High Sensitivity NGS Fragment Analysis Kit with the Fragment Analyzer (Advanced Analytical) following the manufacturers’ protocols (Table 2). The Bioanalyzer and TapeStation concentration estimates were adjusted to comprise the entire library peak, and to exclude fragments likely to be adapter dimers based on their fragment lengths.

**Table 2:** Properties of the quantification methods applied.

Instrument	Supplier	Quantification mechanism	Required vol. input DNA	Quantitative range^A^	No. of samples tested simultaneously	Time estimation	Reagent costs per sample (US$)
NanoDrop	Thermo Fisher Scientific	UV spectro-photometry	1 µL	2–3, 700 ng/uL	1	30 sec per sample	<0.5
Qubit^B^	Thermo Fisher Scientific	Fluoroscence spectroscopy	1–20 µL	10 pg/µL–100 ng/µL	1	1 min per sample	0.7
Bioanalyzer^B^	Agilent Technologies	Electrophoresis	1 µL	5–500 pg/uL	1–11	60 min for ≤11 samples	8.1
TapeStation^B^	Agilent Technologies	Electrophoresis	2 µL	10–1000 pg/uL	1–16	60 min for ≤16 samples	3.6
GX Touch^B^	PerkinElmer	Electrophoresis	1–20 µL	5–5, 000 pg/µL	1–24	≤96 samples in 2.5 hours	2.3
Fragment Analyzer	Advanced Analytical	Capillary electrophoresis	2 uL	50–500 pg/µL (fragment) 50–5000 pg/uL (smear)	≤96, ≤288, or ≤480^c^	1.5 hours for ≤12, ≤48, or ≤96 samples^C^	2.3
ABI7500	Thermo Fisher Scientific	qPCR SYBR Green GeneRead	2 µL	0.00083–8.3 pM^D^	≤96	≤96 samples in 2.5 hours	3.0
ABI7500	Thermo Fisher Scientific	qPCR TaqMan IonLibQuant	2 µL	0.068–6.8 pM^D^	≤96	≤96 samples in 2.5 hours	5.9

^A^According to instrument data sheets; ^B^For High Sensitivity kits; ^C^Depends on whether 12-, 48-, or 96-capillary plates are applied; ^D^The range of the standard curve used for the assay was set as quantitative range.

Precision ID and Ion Xpress fragment libraries as well as dilutions of the Ion Torrent oligos were further quantified with the ABI7500 instrument and the HID Real-Time PCR Analysis Software v1.2 (Thermo Fisher Scientific). The GeneRead Library Quant Kit (Qiagen) or the Ion Library Quantitation Kit (IonLibQuant) (Thermo Fisher Scientific) were used following the recommendations of the manufacturers. qPCR molar concentrations were converted into concentrations in pg/µL using fragment lengths of 203 and 280 bp for Precision ID Identity Panel and Ion Xpress fragment libraries, respectively.

All quantifications were performed in duplicates. The averages were used for evaluation of the quantification methods. Two-sided Friedman’s tests^20^ with Bonferroni correction were performed using the “friedman.test.with.post.hoc” application in R 3.3.0 to test for significant differences in concentration estimates among the quantification methods. Due to the differences in library concentrations, the data were not expected to be normally distributed. Two tests were done: one including all the libraries (n = 30) and excluding the qPCR quantifications, and one excluding the NimbleGen libraries (n = 24), but including quantifications with all methods. P values for comparisons among quantifications with NanoDrop, Qubit, Bioanalyzer, GX Touch, TapeStation, and Fragment Analyzer were obtained from the first test with a level of significance at α = 0.0033 after Bonferroni correction. P values for comparisons to the real time-PCR quantifications were obtained from the second test with α = 0.0018 after Bonferroni correction.

### Correlation between TapeStation and ABI7500 measurements

Three Precision ID Ancestry Panel libraries were diluted 2×, 4×, 8×, 16×, 32×, and 64×. Each dilution was quantified three times with the TapeStation and the ABI7500 instruments using the IonLibQuant assay. The means of each of the triplicate measurements were used for further analysis. A fragment length of 207 bp was used to convert molar concentrations into weight concentrations. The relationship between the TapeStation and the ABI7500 concentration estimates were tested using linear regression analyses with the “lm” application in R 3.3.0 and the square of the Pierson correlation coefficient. For each library, the limit of quantification (LOQ) was determined as 10 times the standard deviation of the regression line’s ordinate intercept divided by the slope of the regression line as previously described^21^.

### Sequencing coverage analysis

Thirty-five Precision ID Ancestry Panel libraries were sequenced in pools containing 18 libraries (one library was not used in this study), which had been pooled in equimolar concentrations according to Qubit, TapeStation, or ABI7500 (IonLibQuant assay) quantifications. Each pool had a total library concentration of 50 pM. Template preparation consisting of emulsion PCR, enrichment of beads containing template, and chip loading was performed with the Ion Chef instrument and the Ion PGM Hi-Q Chef Kit according to the manual (Thermo Fisher Scientific). The loaded sequencing chips were placed onto the Ion PGM™ instrument (Thermo Fisher Scientific) together with Ion PGM Hi-Q Chef 400 Supplies Kit (Thermo Fisher Scientific) and sequenced for 500 cycles according to the manual. Sequence analysis was done using the Torrent Suite Software v.4.4.2 with the HID_SNP_GenoTyper v. 4.2 plugin. Linear regression and two-sided F test were performed using the “lm” application in R 3.3.0 to test for correlations between concentration estimates and library coverage using data from all 35 libraries and α = 0.05.

### Data availability

All data generated or analysed during this study are included in the article and the supplementary information.

## Results

### Comparison of concentration estimates

**Figure 1:**
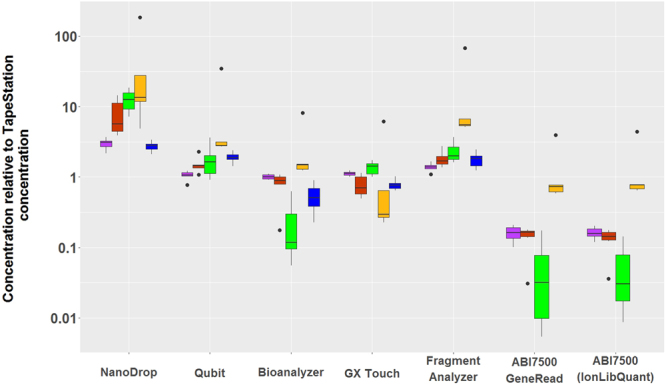
Relative concentration estimates obtained using eight quantification methods. All quantifications were performed in duplicates. The mean was normalised by dividing with the mean of the concentration estimate obtained with the TapeStation instrument. Among the 18 Precision ID Identity Panel libraries, six were categorised as “well PCR amplified” (purple boxplots), six as “adapter dimer rich” (red boxplots), and six as “PCR inhibited” (green boxplots). Ion Xpress fragment libraries and NimbleGen capture libraries are shown in yellow and blue plots, respectively. The NimbleGen capture libraries were not quantified by qPCR, since the qPCR assays targeted Ion Torrent adapters. The lower and upper limits of the box correspond to the 0.25 and the 0.75 quartiles, respectively, and the median is indicated as a line within the box. The ends of the whiskers correspond to the most extreme data point within 1.5 times the interquartile range from the ends of the box. Outliers are indicated by dots.

Eighteen Precision ID Identity Panel libraries, six low concentration Ion Xpress fragment libraries, and six high concentration NimbleGen capture libraries were quantified using eight quantification methods (Table 3). Among the 18 Precision ID Identity Panel libraries, six were categorized as “well PCR amplified”, six as “adapter dimer rich”, and six as “PCR inhibited” based on the fragment length distributions visualized by the Bioanalyzer instrument and the inhibition of real time-PCR reactions of the target DNA. Figure 1 illustrates the relative differences in the quantification of the 30 MPS libraries. For each sample, the mean of the duplicate quantifications was normalized by dividing the measurement with the mean of the concentration estimate obtained with the TapeStation. The concentration estimates varied statistically significantly between the different methods and could be divided into three groups: 1) Estimates from the NanoDrop, 2) estimates from the electrophoresis instruments and the Qubit, and 3) estimates from the qPCR assays. The NanoDrop gave the highest estimates. They were statistically significantly different from the estimates of the other instruments after correction for multiple testing (p = 4.87*10^−4^, p = 1.11*10^−16^, p = 2.65*10^−13^, and p = 1.43*10^−14^ for comparison to Qubit, Bioanalyzer, GX Touch, and TapeStation, respectively), except for the comparison between the NanoDrop and the Fragment Analyzer (p = 0.128). The majority of the tested libraries had concentrations below the quantitative range of the NanoDrop (Table 2), which may explain the high concentration estimates and the large variations among them. The estimates of the four different electrophoresis instruments and the Qubit were similar, although the estimates of the Fragment Analyzer were, on average, 1.3-2.7 times higher than those of the other instruments in this group. The Fragment Analyzer measurements were statistically significantly different from the estimates of the Bioanalyzer, GX Touch, and the TapeStation after correction for multiple testing (p = 4.76*10^−9^, p = 8.67*10^−6^, and p = 9.00*10^−7^, respectively). Similar results were obtained with the ABI7500 qPCR instrument with the GeneRead and the IonLibQuant kit. The ABI7500 quantification results were lower than those obtained with the other methods, and they were statistically significantly different from the estimates of the NanoDrop, the Qubit, and the Fragment Analyzer after correction for multiple testing (p = 0.00/p = 3.33*10^−16^ comparing to GeneRead/IonLibQuant, p = 1.10*10^−7^/p = 1.02*10^−6^, and p = 9.25*10^−13^/p = 4.49*10^−11^, respectively). The variation in the relative concentration estimates of the six “PCR inhibited” libraries was large (Fig. 1), especially for the qPCR estimates. This indicates that PCR inhibition most likely influenced the qPCR reactions.

**Table 3:** Concentration measurements of MPS libraries (pg/µL).

Library	NanoDrop	Qubit	Bioanalyzer	GX Touch	TapeStation	Fragment Analyzer	qPCR GeneRead	qPCR IonLibQuant
Well amplified	4,685	1,685	2,000	2,560	2,170	2,361	218	260
Well amplified	5,940	2,330	2,072	2,355	1,905	2,740	281	281
Well amplified	3,530	1,495	1,433	1,430	1,370	2,017	246	199
Well amplified	4,090	1,525	1,438	1,440	1,305	2,171	255	263
Well amplified	4,375	1,230	1,087	1,190	1,185	1,565	155	201
Well amplified	4,455	1,415	1,318	1,540	1,360	1,785	283	255
Adapter dimer rich	27,250	2,035	1,922	1,170	1,895	2,810	259	335
Adapter dimer rich	2,390	882	498	485	618	841	99	76
Adapter dimer rich	2,315	526	386	195	395	811	67	68
Adapter dimer rich	2,625	708	374	265	475	786	81	69
Adapter dimer rich	3,490	1,230	905	920	845	1,447	151	120
Adapter dimer rich	2,535	412	32	205	181	496	6	7
PCR inhibited	5,130	317	17	535	309	619	2	5
PCR inhibited	3,210	678	152	380	377	652	38	38
PCR inhibited	4,420	556	33	505	371	599	2	3
PCR inhibited	3,250	417	284	470	456	901	80	65
PCR inhibited	2,660	740	26	320	205	604	7	8
PCR inhibited	4,410	498	27	360	240	884	7	6
Fragment	1,240	334	157	30	105	704	77	82
Fragment	635	365	201	40	131	702	101	101
Fragment	3,230	314	150	0	116	641	71	79
Fragment	1,190	246	110	20	89	454	52	57
Fragment	900	170	40	30	5	331	19	22
Fragment	1,340	198	26	0	0	296	11	19
Capture	77,075	48,000	14,024	20,340	26,050	35,327	N/A	N/A
Capture	86,780	54,900	9305	21, 145	25,750	48,797	N/A	N/A
Capture	48,065	43,500	16,495	18,535	18,250	44,891	N/A	N/A
Capture	56,705	40,400	17,291	15,490	23,050	46,448	N/A	N/A
Capture	96,920	62,400	7382	24,420	32,800	57,615	N/A	N/A
Capture	54,690	36,900	12,282	16,610	25,850	32,008	N/A	N/A

Averages of duplicate measurements are listed. “Well amplified”, “Adapter dimer rich”, and “PCR inhibited” libraries are amplicon libraries.

### Sensitivity testing

**Figure 2:**
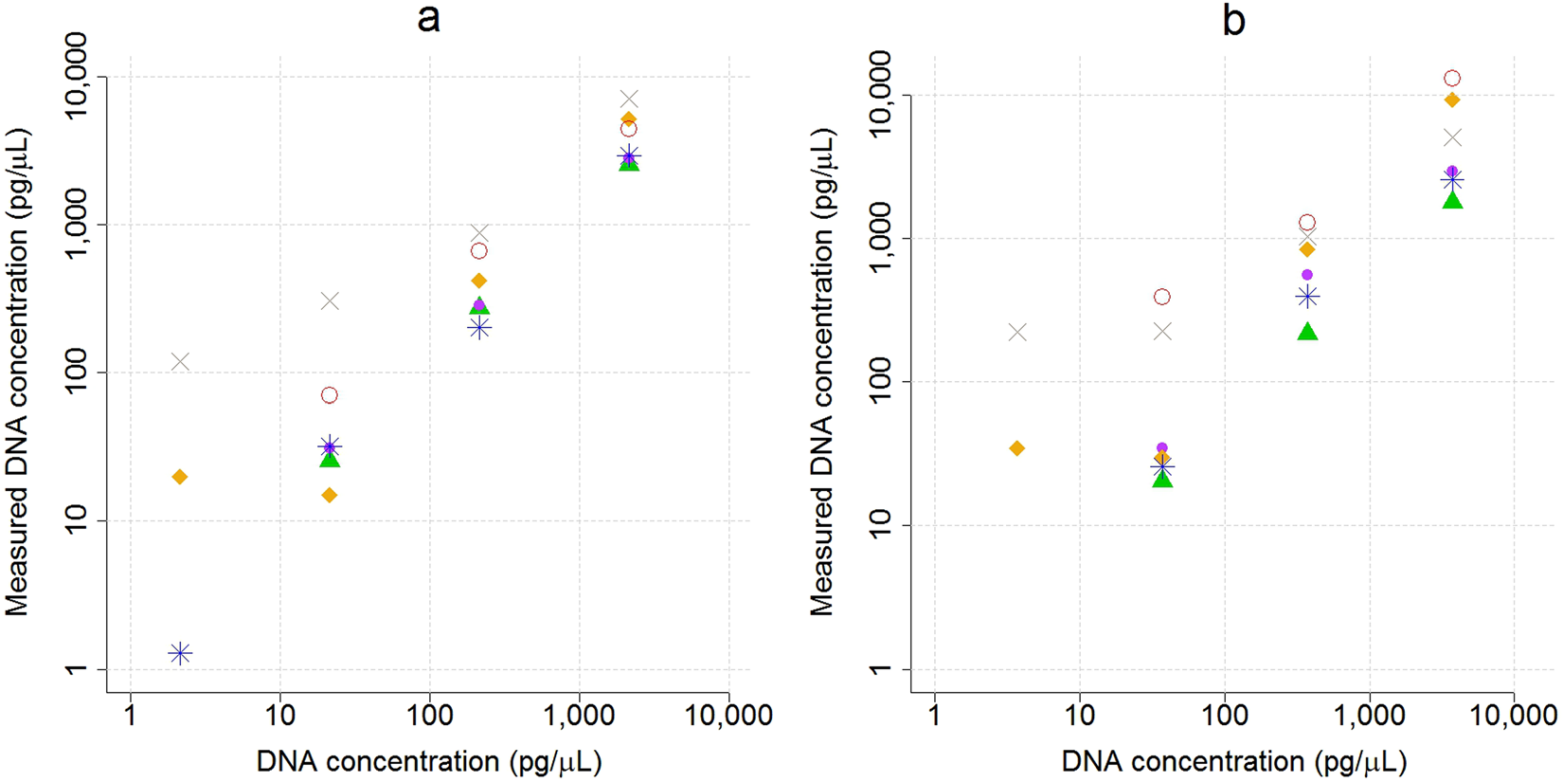
Quantification of synthetic double-stranded oligos. Four dilutions of two synthetic double-stranded oligos consisting of either the Ion Torrent “A” and “P1” adapter sequences (**a**) or the Illumina “i7” and “i5” adapter sequences (**b**) were quantified in duplicate with the NanoDrop (), Qubit (), Bioanalyzer (), GX Touch (), TapeStation (), and Fragment Analyzer (). The mean of the measured oligo concentrations were plotted against the concentrations given by the oligo supplier.

**Figure 3:**
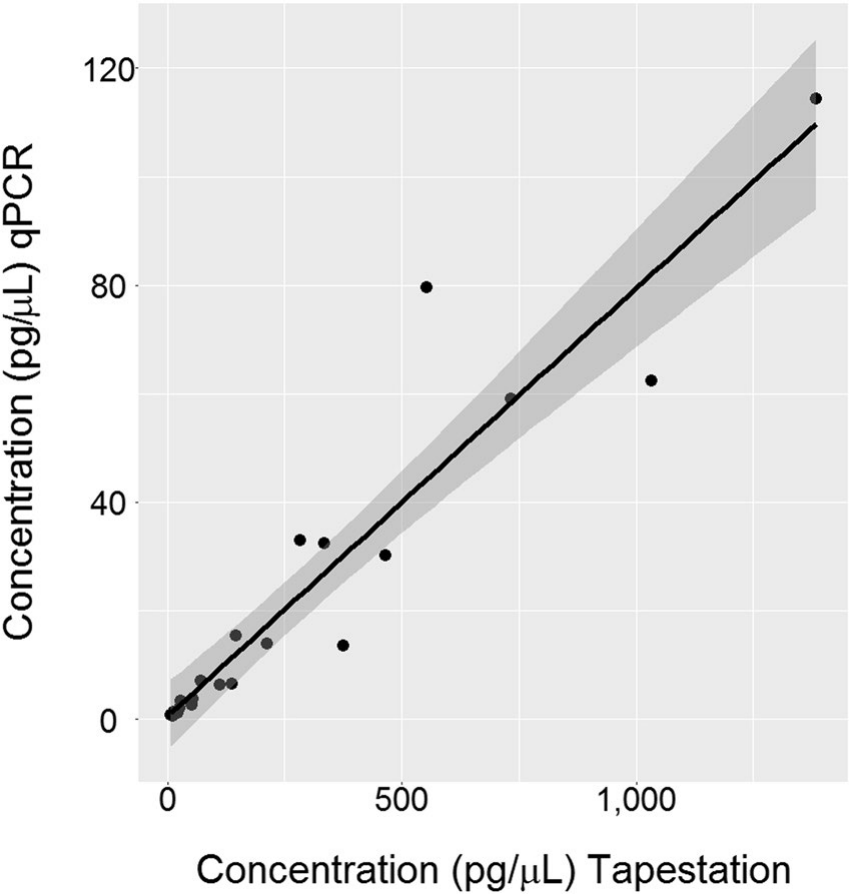
Linear regression analysis of quantifications using the TapeStation and the ABI7500 qPCR instrument with the IonLibQuant assay. Three Precision ID Ancestry Panel libraries were diluted 2×, 4×, 8×, 16×, 32×, and 64×. The dilutions were quantified in triplicate. Means of the triplicates were used for the analysis. The line indicates the linear regression model (y = 0.08× + 0.6, R^2^ = 0.89). The grey area indicates the 95% confidence interval.

**Figure 4:**
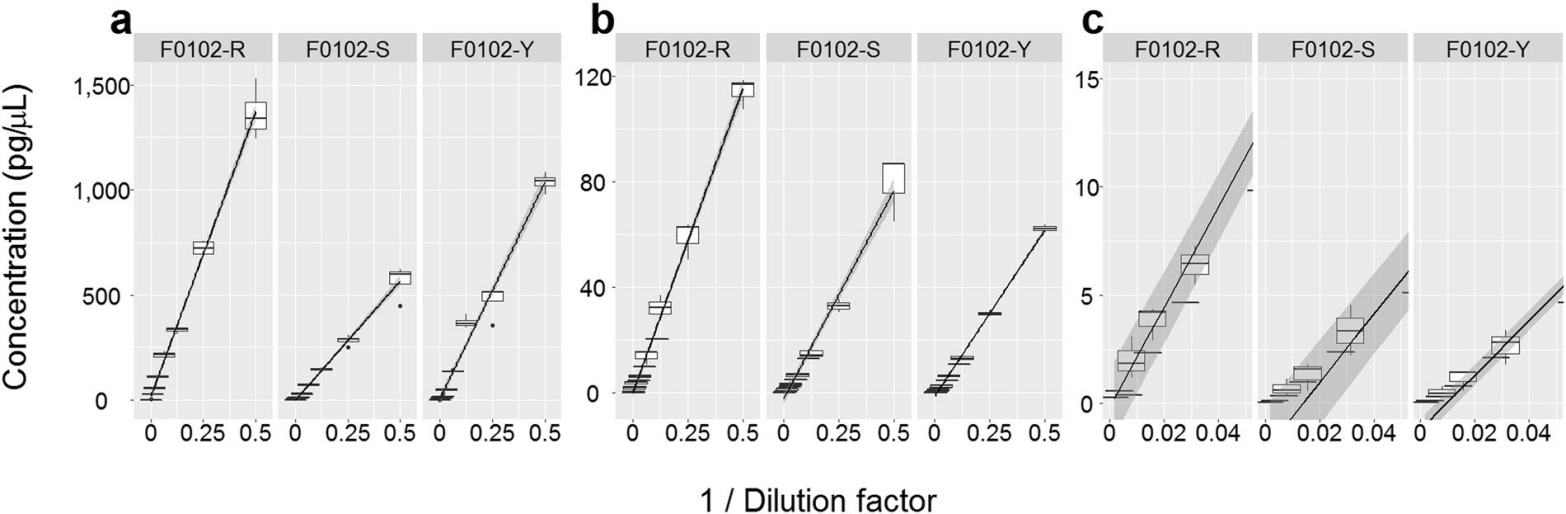
Quantification of dilution series of three Precision ID Ancestry Panel libraries using the TapeStation instrument (**a**) and the ABI7500 qPCR instrument with the IonLibQuant assay (**b**,**c**). The c plot is a zoom of the b plot. The libraries were diluted 2×, 4×, 8×, 16×, 32×, and 64×. Boxplot properties are explained in the legend of Fig. 1. Lines represent linear regression lines. Grey areas represent 95% confidence intervals.

The sensitivities of the different quantification methods were tested by dilution series of dsDNA oligos (Fig. 2, Supplementary Table 2). The TapeStation, Bioanalyzer, and Qubit estimates were closest to the concentrations given by the oligo supplier, whereas the NanoDrop, Fragment Analyzer, and the GX Touch overestimated the concentrations. The relative variation of the estimates increased for concentrations below 40 pg/µL, and concentrations below 20 pg/µL were rarely detected. The dsDNA oligos could neither be amplified by the GeneRead nor the IonLibQuant assay on the ABI7500 qPCR instrument. The sensitivity of the qPCR was compared to that of the TapeStation by quantifying Precision ID Ancestry Panel libraries (Fig. 3). The two quantification methods were linearly correlated to each other for a wide range of concentrations (R^2^ = 0.89, P = 2.09*10^−10^). The linear model indicated that the concentration estimates of the qPCR were on average 12.5 times lower than the TapeStation estimates. The ordinate-intercept of the linear model, corresponding to the limit of quantification, was on average 92.6 ± 34.5 and 0.053 ± 0.037 pg/µL for the TapeStation and the ABI7500 qPCR (IonLibQuant assay), respectively (Fig. 4). The limit of quantification of the qPCR assay corresponded to 0.42 pM, which is one magnitude larger than the lower limit of the standard curve used for the IonLibQuant assay (Table 2).

### Correlation between concentration estimates and read counts

**Figure 5:**
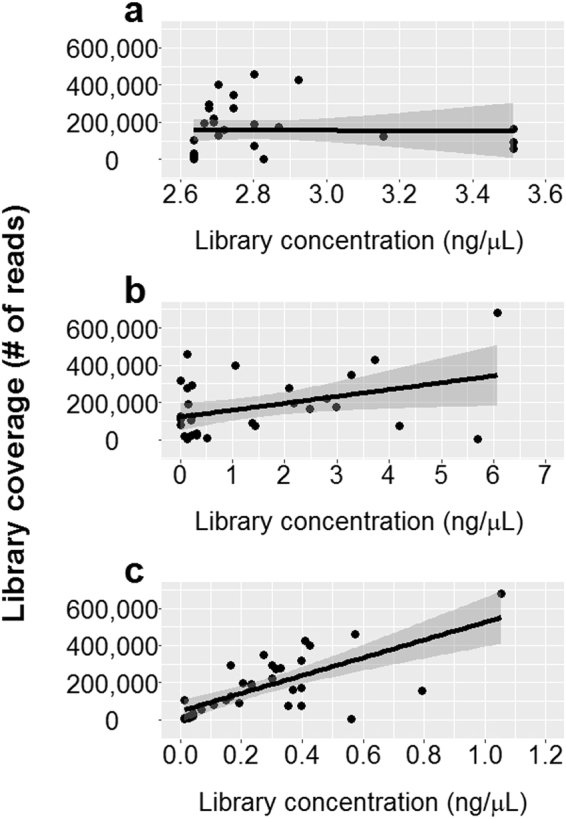
Correlations between library concentration estimates and library coverage. A total of 35 Precision ID Ancestry Panel libraries were quantified prior to sequencing using the Qubit (**a**), TapeStation (**b**), or ABI7500 qPCR (**c**) instrument. The ABI7500 was used in combination with the IonLibQuant assay. Linear regression lines (black line) are plotted with 95% confidence interval (grey area). No correlation was observed between concentration estimates and coverage when using Qubit (R^2^ = 7.4*10^−2^, p = 0.114) or TapeStation (R^2^ = 6.7*10^−3^, p = 0.651), while the correlation obtained with qPCR was R^2 = ^0.49 and p = 2.53*10^−6^.

The correlations between the total number of reads for libraries and the library quantification estimates were analyzed by quantifying 35 Precision ID Ancestry Panel libraries with the Qubit, TapeStation, and ABI7500 qPCR (IonLibQuant assay) instruments prior to sequencing with the Ion Torrent platform (Fig. 5). Using linear regression, a weak correlation was found between the qPCR concentration estimates and the library coverage (R^2^ = 0.49, p = 2.53*10^−6^). However, the library coverage had no correlation with the TapeStation (R^2^ = 6.7*10^−3^, p = 0.651) or the Qubit measured concentrations (R^2^ = 7.4*10^−2^, p = 0.114).

## Discussion

qPCR assays seemed to be the best choice for accurate MPS library quantification. The qPCR assays were the most sensitive quantification methods and the quantification estimates were better correlated with the library coverage of the downstream sequencing reaction. qPCR estimates the number of amplifiable target molecules in the library. Since the clonal amplification step (emulsion PCR or bridge PCR) of MPS is also a PCR reaction^22,23^, this may explain why qPCR concentration estimates are more accurate in predicting the total number of reads of a library. The IonLibQuant and the GeneRead qPCR assay tested here gave very similar concentration estimates (Table 3 and Fig. 1) even though the IonLibQuant assay is TaqMan based and the GeneRead assay is based on SYBR Green detection. A similar conclusion was made in a recent study of real-time quantification methods (Dang *et al*., 2016). The reagent cost of the GeneRead assay was similar to those of most of the electrophoresis assays, and half the price of the IonLibQuant assay (Table 2). The qPCR assays were 5–10 times more costly than the spectrophotometric assays, and they were more time consuming than all other quantification methods (Table 2). More hands-on time and a higher price for more accurate quantification may be preferred compared to a higher risk of large variations in library coverage, especially in clinical and forensic genetic laboratories. The cost of the sequencing reagents and re-sequencing of samples are much higher than the differences in costs between the various quantification methods described here. However, if some variations in library coverage are acceptable, the Qubit fluorometer provided an inexpensive, easy, and fast way of performing DNA quantifications.

The Qubit instrument gave concentration estimates close to those of the electrophoresis-based instruments when quantifying chemically synthesized dsDNA oligos (Fig. 2, Supplementary Table 2), but estimated higher DNA concentrations than the electrophoresis-based instruments (TapeStation, Bioanalyzer, and GX Touch) when MPS libraries were quantified (Fig. 1, Table 3). The Qubit device does not differentiate between different lengths of DNA, and thus, primer dimers, adapter dimers, and fragments (gDNA or PCR products) without adapters will be included in the concentration estimates. The electrophoresis-based methods offer visual assessment of the quality of the libraries. Small molecules such as primer and adapter dimers can be eliminated from the concentration estimates, which may explain the different library concentration measurements of the Qubit instrument and the electrophoresis-based methods. However, fragments without ligated adapters can rarely be distinguished from fragments with adapters by electrophoresis and will be included in the concentration estimate. The large differences in the concentration estimates between the qPCR and Qubit/electrophoresis-based methods indicated that a large fraction of the amplicon libraries (AmpliSeq libraries) could not be amplified in the qPCR, most likely because the fragments had only one or no ligated adapter.

The costs and ease of workflow vary among the electrophoresis-based instruments (Table 2). The Bioanalyzer and the TapeStation were easy to set up. However, the two instruments analyzed fewer samples simultaneously than the other electrophoresis instruments. The Bioanalyzer was the most expensive quantification method. The workflow of the Fragment Analyzer was more complex than those of the TapeStation and the Bioanalyzer. However, many samples can be quantified simultaneously with the Fragment Analyzer, and most of the instrument preparations only need to be done once per 24 hours. The GX Touch provides a reusable cartridge. However, cleaning of the chip is laborious, and it is necessary to have an assumption of the library concentration prior to loading of the chip, because the chip is destroyed if it is overloaded.

Bead-based normalization of libraries has been suggested as an alternative to quantification. Previous studies indicated that bead-based normalization resulted in smaller variation in library coverage than sequencing based on library quantification with e.g. NanoDrop and Bioanalyzer, and subsequent pooling of the libraries in equal molar ratios^24,25^. Commercial bead-based normalization kits are available for both Illumina and Ion Torrent libraries. Beads are added to each library, and library molecules bind to the beads until the beads are saturated. Unbound libraries are discarded, and each library will be represented by the number of bead-bound molecules. Reagents for Illumina bead-based normalization are included with the library kits, which does not cause additional expenses, whereas Ion Torrent bead-based normalization reagents must be purchased in addition to the library kits. This will cause an additional price of app. US$6 per library (Thermo Fisher Scientific). However, the Ion Library Equalizer Kit can be used for all Ion AmpliSeq, Ion Plus Fragment, and Ion Xpress Plus Fragment libraries. Whether bead-based normalization results in smaller library coverage variation than quantification and subsequent normalisation should be addressed in future studies. In a recent study, the genotype reproducibility and the concordance were higher for MPS with qPCR quantification than with bead-based normalization^26^. Sequencing has also been suggested as a way of accurately quantifying MPS libraries, e.g. using the MiSeq sequencer for quantifying libraries for later sequencing on the HiSeq.^7,27^. However, this is a high cost solution.

In conclusion, the specific needs of the laboratory experiment determine which concentration measuring and normalization method is the most beneficial. For instance, if similar sample coverage for all samples typed in the same experiment is a requirement, or the sample throughput per experiment must be maximised, qPCR quantification of the libraries seems to be the best solution. Faster and cheaper quantification methods may be useful if the purpose is to identify libraries with too low DNA concentrations to be successfully sequenced. These samples may be excluded from the library pool, and re-typing of the samples can be initiated immediately. In some workflows, library quantification may be left out entirely if the downstream analysis of the sequencing results is sufficiently stringent to prevent misinterpretation, and if it is acceptable to delay re-typing of the sample(s).

## Electronic supplementary material

Supplementary material
