# Supplementary material for: Quantification of massively parallel sequencing libraries – a comparative study of eight methods
Source: Sci Rep. 2018 Jan 18;8:1110. doi: 10.1038/s41598-018-19574-w (PMC5773690; doi:10.1038/s41598-018-19574-w)
Supplement: Supplementary file 1 — Supplementary material [file 41598_2018_19574_MOESM1_ESM.pdf]

Supplementary information

"Quantification of massively parallel sequencing libraries – a comparative study of eight methods"

Christian Hussing, Marie-Louise Kampmann, Helle Smidt Mogensen, Claus Børsting, Niels Morling

Section of Forensic Genetics, Department of Forensic Medicine, Faculty of Health and Medical Sciences, University of Copenhagen, DK-2100 Copenhagen, Denmark

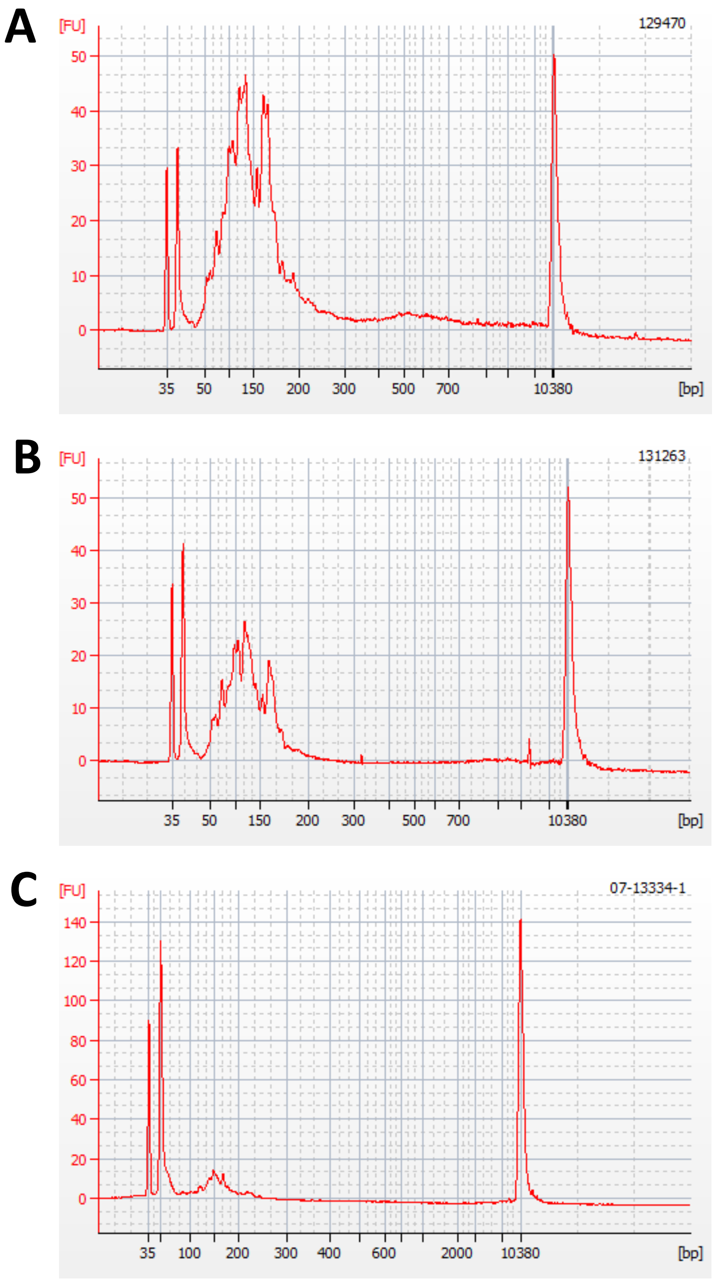

Supplementary Fig. 1. Electrophoresis peak height distribution of a well-amplified library (a), an adapter dimer-rich library (b), and a PCR-inhibited library (c). The 2100 Bioanalyzer electrophoresis instrument was used.

Supplementary Table 1. dsDNA oligos used in this study.

Adapters comprised in oligo	Length (bp)	Sequence (5’ -> 3’)
Ion Torrent adapters	71	CCATCTCATCCCTGCGTGTCTCCGACTCAGCCACTACGCCTCCGCTTTCCTCTCTATGGGCAGTCGGTGAT
Illumina adapters	121	AATGATACGGCGACCACCGAGATCTACACTCTTTCCCTACACGACGCTCTTCCGATCTCTAGCCTTCTCGTGTGCAGACTTGAGGTCAGTGTAGTGCTAGAGCATACGGCAGAAGACGAAC

Supplementary Table 2. Concentration measurements of four dilutions of two oligos with the same sequences as those of the Ion Torrent "A" and "P1" adapters and the Illumina "i7" and "i5" adapters, respectively (pg/µL).

Oligo	Concentration[A]	NanoDrop	Qubit	Bioanalyzer	GX Touch	TapeStation	Fragment Analyzer
Ion Torrent adapters	2,150	4,430	2,530	2,808	5,215	2,945	7,138
	215	665	275	285	420	203	886
	21.5	70	25	31	15	32	305
	2.15	0	0	0	20	1	120
Illumina adapters	3,720	13,075	1,790	2,972	9,365	2,605	5,068
	372	1,285	218	557	840	397	1,043
	37.2	390	20	35	30	26	228
	3.72	0	0	0	35	0	222

Average of duplicate concentration measurements. [A]Concentrations from oligo suppliers.
